
JOURNAL INFORMATION
==============================
NLM Title Abbreviation: Anim Front
Journal ID: Anim Front
NLM Journal ID: 101754918
Journal ID: af

Animal Frontiers: The Review Magazine of Animal Agriculture

ISSN: 2160-6056
EISSN: 2160-6064
Publisher: Oxford University Press

ARTICLE INFORMATION
==============================
PMCID: PMC6952861
PMID: 32002204
DOI: 10.1093/af/vfz045
Article ID: vfz045
Article version: 1
Subjects: Society News

American Society of Animal Science

Publication date: 2020 Jan
Electronic publication date: 2020 Jan 10
Volume: 10
Issue: 1
First page: 59
Last page: 59
Copyright: Copyright © 2020 American Society of Animal Science
Copyright year: 2020
License: This is an Open Access article distributed under the terms of the Creative Commons Attribution License (http://creativecommons.org/licenses/by/4.0/), which permits unrestricted reuse, distribution, and reproduction in any medium, provided the original work is properly cited.
License URL: https://creativecommons.org/licenses/by/4.0/

Page count: 1

==============================
AMERICAN SOCIETY OF **ANIMAL SCIENCE**

The American Society of Animal Science fosters the discovery, sharing and application of scientific knowledge for the responsible use of animals to enhance human life and well-being.

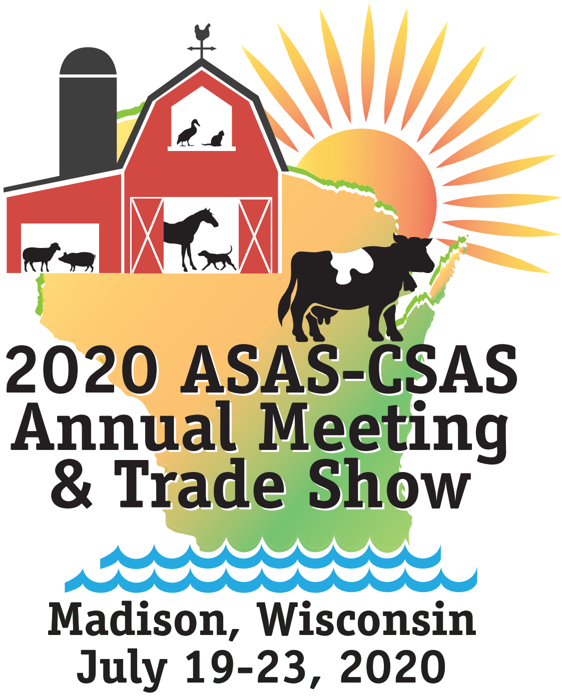

**Nestled between lakes Mendota and Monona, Madison is one of only two major U.S. cities built on an isthmus.**

Greater Madison is an eclectic mix of cultures, from college town to capital, from single students to families large and small. We've got a small town feel, and big city amenities. It's no wonder we've earned national notoriety for some of the people and places that contribute to what makes our city so memorable.

Besides boasting natural beauty and outdoor recreation, Madison plays host to stimulating cultural offerings, distinctive restaurants, excellent music venues and unique shopping. Ours is a true college town, and the intellectual offerings of the University of Wisconsin-Madison and surrounding schools attract scholars from around the world.

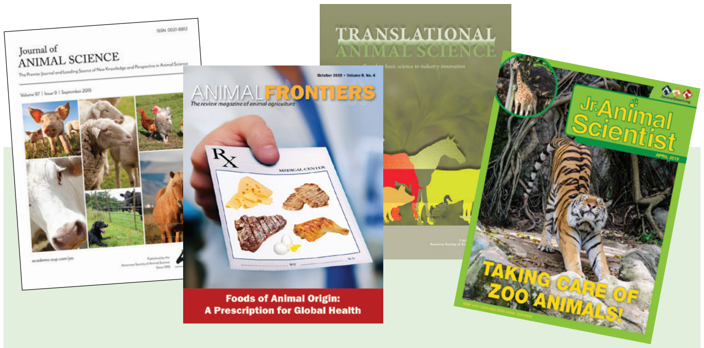

### ASAS Publications

***The Journal of Animal Science***, an official journal of the American Society of Animal Science, publishes research on animal production and genetics, nutrition, physiology and the utilization of animal products.

***Translational Animal Science***, an official journal of the American Society of Animal Science, encompasses a broach scope of research topics in animal science, focusing on translating basic science to innovation.

***Animal Frontiers***, an official journal of the American Society of Animal Science, publishes discussion and position papers that present several international perspectives on the status of a high-impact, global issues in animal agriculture.

***Junior Animal Scientist***, an official magazine of the American Society of Animal Science, offers elementary or middle school students a way to learn the real science behind pets, zoo animals and farm animals. A membership is offered and encompasses both the magazine and access to a special part of AnimalSmart.org. Sign your Junior Animal Scientist up today!

To access articles and learn more about ASAS Publications, visit **asas.org**.

### FUTURE MEETINGS

Southern Section Meeting — Jan. 25-28, 2020

Midwest Section Meeting — March 2-4, 2020

Western Section Meeting — June 16-18, 2020

ASAS-CSAS Annual Meeting — July 19-23, 2020

doi: 10.1093/af/vfz045
